# The Last Mile in Polio Eradication: Program Challenges and Perseverance

**DOI:** 10.3390/pathogens13040323

**Published:** 2024-04-15

**Authors:** Rocio Lopez Cavestany, Martin Eisenhawer, Ousmane M. Diop, Harish Verma, Arshad Quddus, Ondrej Mach

**Affiliations:** Polio Eradication, World Health Organization, 1202 Geneva, Switzerland; eisenhawerm@who.int (M.E.); diopo@who.int (O.M.D.); vermah@who.int (H.V.); quddusa@who.int (A.Q.); macho@who.int (O.M.)

**Keywords:** poliomyelitis, Global Polio Eradication Initiative, outbreak response, immunization, vaccine-derived poliovirus

## Abstract

As the Global Polio Eradication Initiative (GPEI) strategizes towards the final steps of eradication, routine immunization schedules evolve, and high-quality vaccination campaigns and surveillance systems remain essential. New tools are consistently being developed, such as the novel oral poliovirus vaccine to combat outbreaks more sustainably, as well as non-infectiously manufactured vaccines such as virus-like particle vaccines to eliminate the risk of resurgence of polio on the eve of a polio-free world. As the GPEI inches towards eradication, re-strategizing in the face of evolving challenges and preparing for unknown risks in the post-certification era are critical.

## 1. Introduction

From the earliest believed documentation of polio c. the 1400s BC through a depiction on an Egyptian stele, it was not until the mid-19th century that the first systematic investigation of polio was conducted. The first records of large-scale polio epidemics began appearing in North America and Europe; only in the mid 20th century was it possible to confirm global poliovirus spread, which killed or paralyzed over half a million people every year [1].

The development of polio vaccination in the 1950s and 1960s was a turning point to begin controlling transmission and reducing the burden of disease. The inactivated poliovirus vaccine (IPV), containing formalin-killed virus, was developed by US physician Jonas Salk and licensed for use in 1955. Shortly after, the oral poliovirus vaccine (OPV), containing live-attenuated poliovirus, was developed by Polish American microbiologist Albert Sabin. IPV and OPV induce a strong humoral response capable of protecting the vaccinated individual; however, only the latter has an established role in inducing mucosal intestinal immunity, which is essential to block viral transmission, making it a necessary tool for eradication [2]. Each vaccine type has its own unique set of advantages and limitations; both continue to play an essential role in polio eradication to this day. 

The impact of immunization was immediate; however, there was a geographical disparity in vaccine choice. Countries adopting IPV were largely confined to high-income settings due to the cost, required skill, and hygiene measures associated with injectable vaccines. Easily administered and accessible, OPV was the ideal candidate for use in lower-income settings for routine immunization or outbreak control. Independent of vaccine choice, the prevalence of low vaccination coverage in low-income and high-risk settings created a widening health divide, with the burden of disease shifting to areas in Africa and Asia with weak health and hygiene infrastructure.

Polio eradication later led by the Global Polio Eradication Initiative (GPEI) was launched in 1988 with the aim to eradicate poliomyelitis caused by poliovirus by 2000. This motivation followed smallpox eradication, one of the greatest successes in public health history, paving the way for polio as the second candidate for eradication. The GPEI coordinates global, regional, and country-level interdisciplinary actions towards eradication of polioviruses. The partnership is led by six agencies (the WHO, CDC, Rotary, BMGF, Gavi, and UNICEF) working closely with countries, donors, foundations, NGOs, research partners, and vaccine manufacturers. The GPEI strategy includes strengthening routine immunization (RI), implementing supplementary immunization activities (SIAs) at different scales for large outbreaks or local emergences, and rapid detection and response to transmission via clinical (acute flaccid paralysis, AFP) and environmental surveillance (ES) [3]. This review discusses the status of polio eradication, the latest advancements, and the next (and final) steps needed to achieve eradication. We also discuss what a polio-free world would look like and whether polio vaccination can—or should—ever be stopped once eradication is achieved. 

## 2. Current Status of Polio Eradication

### 2.1. Global Polio Epidemiology

Since the start of the global polio eradication program in 1988, there has been a >99% reduction in polio cases caused by Wild Polioviruses (WPVs) (from >350,000 to just 12 WPV cases in two countries in 2023) and 20 million paralysis cases prevented attributed to vaccination [4,5]. Two out of the three WPV serotypes have been certified as eradicated namely WPV2 in 2015 and WPV3 in 2019, with the last detections in 1999 in India and 2012 in Nigeria, respectively [6,7]. There are only two endemic countries left with WPV1 transmission, namely Afghanistan and Pakistan. Nigeria was the latest former endemic country, with the last WPV1 case reported in 2016 [8,9].

WPV is not the only poliovirus that can cause paralytic poliomyelitis. On rare occasions, the live-attenuated polioviruses from OPV can mutate, circulate in populations with inadequate immunization levels for an extended period, and revert into circulating vaccine-derived polioviruses (cVDPVs) capable of inducing paralysis and causing outbreaks [10]. Clinically and epidemiologically, WPV and cVDPV are indistinguishable. Both readily transmit in poorly immunized communities, cause paralytic outbreaks, and require the same level of programmatic response. Since 2012, paralytic poliomyelitis cases caused by cVDPV have surpassed those caused by WPV. Poliovirus transmission, both by WPV and cVDPV, has been declared as a Public Health Emergency of International Concern (PHEIC) since 2014 [11]. Polio is the longest-standing PHEIC and the only one declared by the World Health Organization at present (January 2024). 

**Figure 1:**
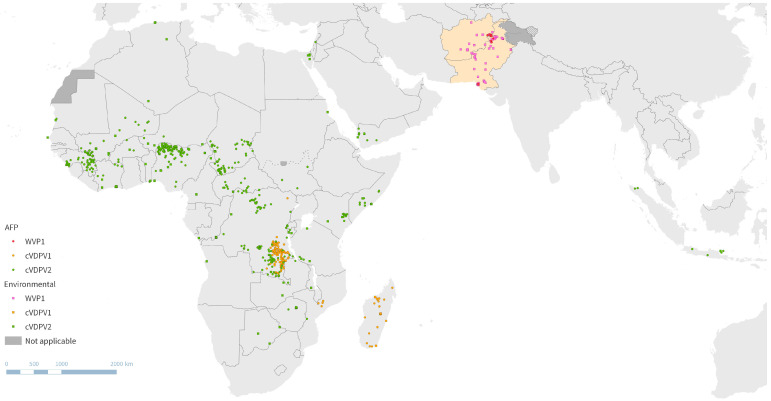
2023 global overview of positive poliovirus isolates, including types 1, 2, and 3 wild poliovirus (WPV) and circulating vaccine-derived poliovirus (cVDPV). Clinical acute flaccid paralysis (AFP) and environmental surveillance (ES) poliovirus detections are depicted. On the left, there is a per-country list of the latest detections of AFP and ES. Data source: from WHO HQ as of 27 February 2024.; figure adapted from internal WHO HQ figures; map creation 5 April 2024; map produced by WHO GIS Centre for Health, DNA/DDI. Disclaimer: The designations employed and the presentation of the material in this publication do not imply the expression of any opinion whatsoever on the part of the WHO concerning the legal status of any country, territory, city, or area or of its authorities or concerning the delimitation of its frontiers or boundaries. Dotted and dashed lines on maps represent approximate border lines for which there may not yet be full agreement.

**Figure 2:**
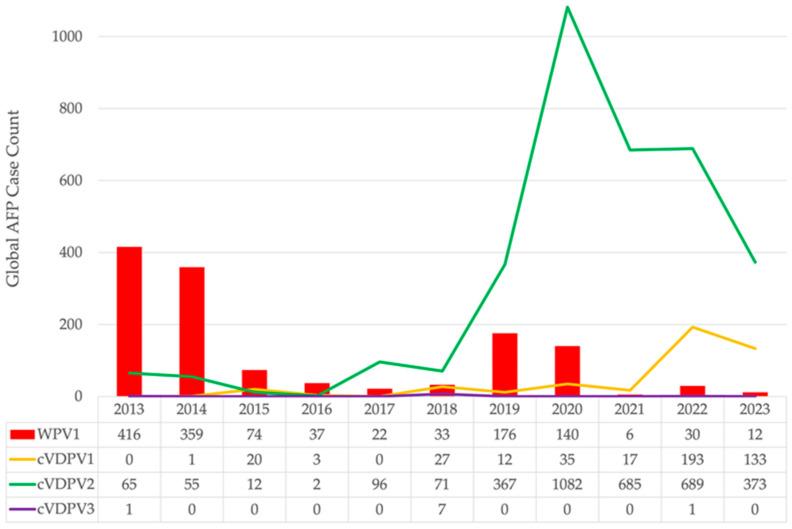
Case count of global clinical AFP poliovirus cases in the past decade (2013–2023) including WPV1 and cVDPV types 1, 2, and 3. Data from WHO HQ as of 27 February 2024. Abbreviations: cVDPV, circulating vaccine-derived poliovirus; WPV, wild-type poliovirus; AFP, acute flaccid paralysis.

In 2023, there were 12 paralytic cases caused by WPV1 globally and 491 paralytic cases caused by cVDPV (133 by type 1, 373 by type 2, and 0 by type 3) (Figure 1) [4,12]. A timeline of global WPV and cVDPV AFP case counts in the last 10 years is depicted in Figure 2. 

WPV1 cases have been largely restricted to one epidemiological block centered along the Pakistan–Afghanistan border with only two genetic clusters (YB3A and YB3C); several historic endemic reservoirs are no longer endemic, including Khyber–Peshawar and the Quetta Block [13]. In 2021, there was a WPV1 circulation in Malawi and Mozambique after importation of the virus from Pakistan. A multi-country vaccination and surveillance response successfully stopped the transmission, and the outbreak has since been contained, with the last detection in August 2022. This is a stark reminder that although the prevalence of endemicity has markedly diminished, the presence of transmission anywhere implies a persistent global risk of importation.

WPV2 had been certified as eradicated in 2015, and OPV2 use was resulting in cVDPV2 cases, setting the stage for tOPV withdrawal [14]. As such, in 2016, there was a globally synchronized switch in routine immunization schedules, replacing trivalent OPV (tOPV) with bivalent OPV (bOPV, containing live Sabin poliovirus serotypes 1 and 3) as part of a phased withdrawal of all Sabin strains from OPV, starting with type 2. Although successfully implemented, the inability to quickly and effectively respond to post-switch cVDPV2 outbreaks and IPV shortages creating a widening type 2 immunity gap resulted in a steep increase in cVDPV2 cases in 2020–2022, the effects of which are still present to date [15].

Until 2021, the majority of the global cVDPV2 burden was in Nigeria; in 2023, eastern DRC, northwestern Nigeria, northern Yemen, and south–central Somalia accounted for >80% of the caseload. These have been referred to as ‘consequential geographies’, identified as continuous drivers of cVDPV emergence and transmission. cVDPV2 was recently detected in regions where poliovirus transmission had long ago been eliminated. There were genetically linked cVDPV2 detections in the UK, Israel, Canada, and the USA [16]. These outbreaks, however, did not progress, and only one paralytic case was detected. The remaining cVDPV2 detections were from environmental samples.

Recent successes in the control of transmission have been due to high-quality SIAs and intensified efforts to access zero-dose or underimmunized children in hard-to-reach or conflict settings. However, the gains are fragile and easily reversible when program quality is not maintained. Even small pockets of transmission can have global implications and slow the final steps towards eradication.

### 2.2. Poliovirus Surveillance

The gold standard for detecting poliovirus is through acute flaccid paralysis (AFP) surveillance supplemented by environmental surveillance of sewage samples, the latter of which is capable of detecting early indicators of silent transmission in a population (i.e., non-paralytic cases). Genomic sequencing analyses determine genetic relationships among polioviruses identified in stool and ES specimens. All collected samples are tested in WHO-accredited laboratories belonging to the Global Polio Laboratory Network (GPLN). 

Ensuring high-quality surveillance through sensitivity and timeliness indicators is critical for outbreak detection and rapid activation of national and program-level responses. The Global Polio Surveillance Action Plan 2022–2024 sets a target of 35 days from paralysis onset or collection of a sewage sample to the notification of a polio detection. However, at present, this target is barely met, mainly due to delays in field-to-lab and inter-laboratory shipment of biological samples [17]. Countries with polio laboratories that can complete all procedures in-house are able to meet this target more easily, as is the case in Pakistan, for example. 

The COVID-19 pandemic negatively affected both AFP and ES surveillance systems [18,19]. However, these were improved in 2021, and by the end of 2022, nearly 80% of priority countries experiencing or at high risk of polio transmission met national AFP targets, and the number of ES sites increased by 31% [20]. Recently, the USA and UK have also increased the number of ES sites due to cVDPV2 detections in 2022 [21,22]. Genetic sequencing allowed for identification of the link between poliovirus detections in the USA, UK, Canada, and Israel.

### 2.3. Challenges and the Current GPEI Polio Eradication Strategy

The current GPEI framework is based on the Polio Eradication Strategy 2022–2026, Delivering on a Promise [3]. The two main goals highlighted in the strategy are (1) permanently interrupting WPV1 transmission in endemic countries and (2) stopping cVDPV transmission and preventing outbreaks in non-endemic countries, both by the end of 2023, with the aim of reaching eradication by 2026. The Independent Monitoring Board (IMB), a panel of external advisors promoting program accountability, analyzed current program performance and the remaining challenges and concluded that goal one is off-track and goal two will be missed for the end of 2023 [23]. The GPEI has amended timelines and forecasts that certification of WPV1 will continue to be achieved in 2026 and that cVDPV2 transmission will be interrupted in 2025 [24].

Geopolitical risks directly impact eradication efforts, particularly through conflict, insecurity, and inaccessibility; these are present in the endemic reservoirs and ‘consequential geographies’ [23]. The deteriorating acute humanitarian crisis in Afghanistan and rising insecurity alongside political instability in Pakistan pose formidable challenges. Although vaccination activities and accessibility in Afghanistan are at their best since 2018, house-to-house vaccination campaigns have been forbidden in southern and northeastern regions of Afghanistan by the Taliban; this could have influenced the recent re-emergence of transmission in Kandahar for the first time in two years [13].

Poliovirus serotype co-circulation increases the complexity of outbreak response in terms of vaccine choice and implementation; eastern DRC has been a strong focal point of cVDPV1 and cVDPV2 co-circulation. In addition to DRC, there are pockets of persistently missed children due to inaccessibility, notably in northwestern Nigeria, south–central Somalia, and the Mehsud Belt in Pakistan. In northern Yemen, there has been an explosive outbreak of VDPV since limited immunization activities have been possible since late 2022. There have been over 200 boycotts of polio vaccination campaigns in Khyber–Pakhtunkhwa, Pakistan. These are only some examples of the formidable challenges that affect global polio eradication efforts; the GPEI continuously reviews and reformats program activities and priorities according to the evolving landscape.

As previously mentioned, the COVID-19 pandemic has significantly affected the polio program. Routine childhood immunization programs, vaccination campaigns (>60 polio SIAs were suspended), and surveillance systems (with gaps and detection lags) were severely disrupted. 

## 3. Closing in on Poliovirus Eradication

### 3.1. Evolving Routine Immunization Schedules

The Strategic Advisory Group of Experts on Immunization (SAGE) is a committee established to advise the WHO on global policies and strategies for vaccines and immunization. As previously mentioned, to eliminate the risk of Sabin type 2-containing vaccines, the SAGE recommended the synchronized withdrawal of tOPV from RI schedules and replacement with bOPV [14]. This meant that only types 1 and 3 antigens were administered via OPV through RI. As such, the SAGE recommended that at least 1 dose of IPV be introduced to bridge the type 2 immunity gap; the recommendation was then extended in November 2020 to at least two doses of IPV [25,26,27]. As of May 2019, all countries have at least one dose of IPV in their RI schedules. However, 41 countries have yet to introduce the second dose, primarily in Africa. 

Due to the delay in IPV manufacturing scale-up after ‘the switch’, several birth cohorts remained naïve to type 2 poliovirus. As a dose- and cost-sparing method, fractional-dose IPV (fIPV) was recommended by SAGE as a viable option for countries to implement in their national immunization guidelines [28]. Clinical trials demonstrate superior immunogenicity of two fIPV doses over one full dose of IPV [29,30]. There are six countries that currently use off-label fIPV administered intradermally in their RI schedules, namely Bangladesh, Cuba, Ecuador, India, Sri Lanka, and Nepal. Serological assessments from Cuba, Ecuador and India demonstrated high immunogenicity of their fIPV immunization schedules [29,31,32].

**Figure 3:**
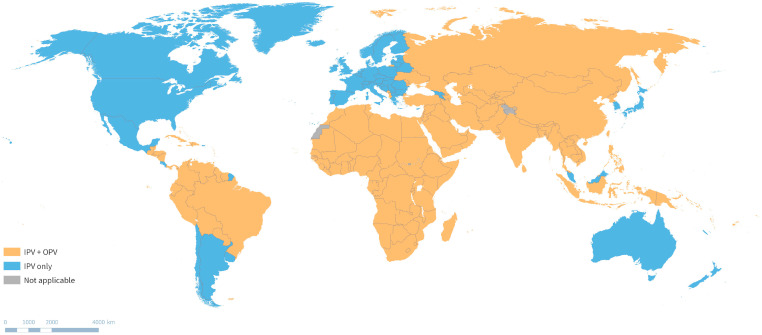
Routine polio immunization schedules of countries using inactivated poliovirus vaccines (IPVs) or IPVs plus oral poliovirus vaccines (OPVs) administered sequentially. Data source: WHO as of December 2023; map creation date 05 April 2024; map production by WHO GIS Centre for Health, DNA/DDI. Disclaimer: The designations employed and the presentation of the material in this publication do not imply the expression of any opinion whatsoever on the part of WHO concerning the legal status of any country, territory, city, or area or of its authorities or concerning the delimitation of its frontiers or boundaries. Dotted and dashed lines on maps represent approximate border lines for which there may not yet be full agreement.

As countries become polio-free, are at reduced risk of importation and transmission, and have high and homogenous RI coverage and good sanitation, there is a shift in RI schedules from using OPV to IPV only. There are 67 countries that have IPV-only RI schedules—primarily industrialized countries (Figure 3) [33]. The use of OPV is incompatible with complete poliovirus eradication, as it contains live virus; therefore, plans are being made for complete cessation of OPV use in RI. bOPV cessation is planned to be implemented one year after certification of eradication of WPV1, provided all (yet-to-be-determined) pre-requisites are met.

Most of the countries using IPV-only RI schedules are using combination vaccines instead of standalone IPV—in most cases, pentavalent or hexavalent vaccines with an acellular pertussis component. Gavi, The Vaccine Alliance, besides their ongoing support for standalone IPV, approved the addition of a whole-cell pertussis hexavalent (wP-Hexavalent) vaccine to its portfolio in June 2023, making it more accessible globally [34]. The wP-Hexavalent vaccine contains six antigens, including the full dose IPV and the pentavalent vaccine antigens, including diphtheria, tetanus, pertussis, hepatitis B, and haemophilus influenza type b. Benefits of a hexavalent vaccine include a reduced number of injections in the RI schedule and higher sustainable IPV coverage. SAGE recommended a 3–4 dose schedule to accelerate and secure polio eradication [35]. The official launch of the hexavalent program was on 1 December 2023, and the first introduction of wP-Hexavalent in any country is expected in mid-2024 [34]. Gradually, more countries are expected to adopt wP-Hexavalent vaccines fulfilling the Gavi criteria, prioritization, and demand–supply dynamics.

### 3.2. New Tools and Strategies: Outbreak Response

Between 2010 and ‘the switch’ in 2016, there were 318 cVDPV2 cases in 15 countries; since OPV2 withdrawal in RI, there has been a ~10-fold increase to 3129 cVDPV2 cases in 41 countries. The majority have been attributed to seedings of VDPV2 following mOPV2 use [36].

nOPV2 is a genetically modified version of mOPV2 designed to increase the genetic stability of the vaccine virus and thereby reduce the chance of reversion to a neurovirulent phenotype, all while maintaining comparable immunogenicity [37]. In November 2020, the WHO Prequalification program recommended nOPV2 for cVDPV2 outbreak response under Emergency Use Listing (EUL) [38]. nOPV2 was the first-ever vaccine to receive an EUL, paving the way for other emergency vaccines. Since the start of the rollout in March 2021, nearly one billion doses of nOPV2 have been administered in 35 countries to date, as of January 2024. nOPV2 manufactured by BioFarma, Indonesia obtained full WHO prequalification in December 2023, making it the first vaccine to reach licensure after having been granted an EUL [39].

**Figure 4:**
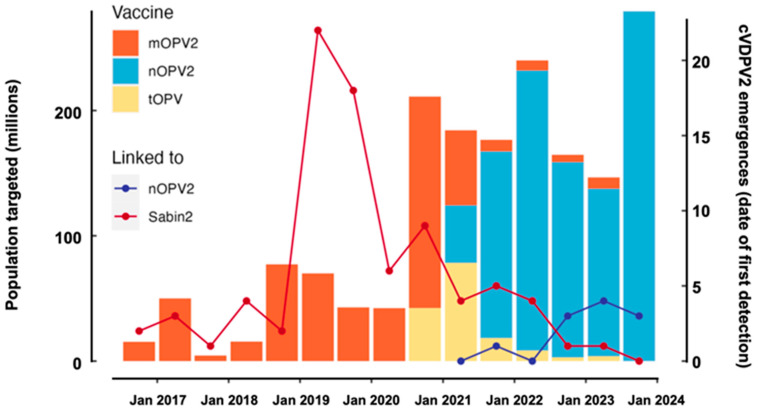
Emergences of cVDPV2 in 2016–2023 after the tOPV-to-bOPV switch linked to OPV use. The figure depicts the use of Sabin OPV (mOPV2 and tOPV) for outbreak response and the introduction of nOPV2 in March 2021. The data are from 1 July 2016, to 31 December 2023. Figure from Bandyopadhyay et al., 2024 [40]. Abbreviations: cVDPV2, type 2 circulating vaccine-derived poliovirus; tOPV, trivalent oral poliovirus vaccine; bOPV, bivalent oral poliovirus vaccine; mOPV2, type 2 monovalent oral poliovirus vaccine; nOPV2, novel type 2 oral poliovirus vaccine.

Field data demonstrate that nOPV2 retains its enhanced genetic stability with a substantially lower rate of reversion and risk of cVDPV2 emergence compared to Sabin OPV2. In fact, the number of recorded global cVDPV2 emergences has decreased markedly since nOPV2 implementation (Figure 4, [40]). By the end 2023, there had been 11 cVDPV2 emergences linked to nOPV2 use—4 originating in DRC, 2 in CAR, 1 in Nigeria, 1 in Egypt, 1 in Botswana, 1 in Cameroon, and 1 in Zimbabwe [41,42]. Modelling suggests that if nOPV2 seeded new emergences at the same rate as Sabin OPV2, 58.4 cVDPV2 nOPV2-derived emergences would be expected, given the total volume of nOPV2 doses used in AFRO (as of 17 November 2023). This results in an estimated 82% reduction in risk of the emergence of cVDPV2 with nOPV2 use compared to Sabin OPV2 [41]. The SAGE recently recommended that the second round of nOPV2 in outbreak response be implemented in a shorter timeframe of 1–4 weeks to ensure timeliness and reduce the risk of emergence [43].

For more efficient outbreak response, SAGE also recommends that IPV (full or fractional dose) be added to OPV in campaigns in areas with persistent poliovirus transmission to more efficiently close the humoral immunity gap and boost mucosal immunity [44].

### 3.3. New Tools and Strategies: Surveillance and Timeliness of Detection

Once a stool or environmental sample is collected for analysis, there is a critical period until the confirmation of a poliovirus isolate, including transport to the laboratory and analysis of the sample. 

The current algorithm used to identify poliovirus from stool specimens and environmental samples involves viral isolation and molecular characterization of the isolates. Sample analysis in the laboratory can take 2–3 weeks, on average, from sample receipt to final sequencing results. The steps in the standard process involve cell culturing for virus isolation, intertypic differentiation by quantitative PCR, and Sanger sequencing to distinguish between vaccine, vaccine-derived, and wild-type polioviruses. 

There are two alternative direct detection technologies currently being assessed, namely (i) direct detection with intratypic differentiation (DD-ITD) and (ii) direct detection nanopore sequencing (DDNS) [45,46]. These methods are faster and safer alternatives compared to standard cell culture regarding containment-related risks associated with growing live viruses. Using direct detection methods aims for increased timeliness in outbreak detection and response. Both DD-ITD and DDNS are being parallel-tested against the current recommended method in several GPLN laboratories, and data are regularly reviewed to assess if the methods meet all evaluation criteria. A decision for implementation into routine use is expected in 2024–2025 after full validation by the GPLN.

While the speed of obtaining laboratory results would be improved by utilizing direct detection methods, an additional impediment to timely case reporting lies within the logistics infrastructure, with sample/isolate transport as the key driver for delays. Village Reach has recently started implementing a program in 15 high-risk countries for poliovirus transmission in Africa to reduce the sample-to-lab transport times via direct engagement with local governments and partners to create tailored plans [47]. The initiative began in 2022 and a positive impact is already noticeable, especially in countries without national laboratory capacity.

## 4. Preparing for a Polio-Free World

### 4.1. Challenges and the GPEI Post-Certification Strategy

The polio Post-Certification Strategy outlines the global technical standards and activities (e.g., containment, vaccination, and surveillance) needed to sustain a polio-free world after global certification of wild poliovirus eradication [48]. The primary risks of a potential resurgence of polio in the post-certification era are the continued use of OPVs, accidental release of poliovirus from a laboratory or vaccine manufacturing facility, and the chronic excretion of VDPVs by persons with primary immunodeficiency (iVDPVs). In addition, bioterrorism involving poliovirus engineering and release is also considered a risk.

Today, live poliovirus is present in four sites, namely humans, the environment, within OPV, or held in designated research or vaccine production facilities. Even when transmission of poliovirus and OPV use is stopped, there is still poliovirus within these facilities, which creates a risk of (intentional or accidental) release into the environment and the re-establishment of transmission. This has happened in the past. Most recently, in the Netherlands, a WPV3 isolate was detected in sewage water originating from an industrial plant; however, no transmission was established [49]. As such, laboratories, vaccine production sites, or any other facility that handles or stores poliovirus must adhere to strict containment guidelines and biosafety requirements. The WHO Global Actional Plan for Poliovirus Containment, recently updated in July 2022 (GAPIV), is the chief reference document for poliovirus containment measures [50].

Finally, a less understood risk is that of iVDPVs (VDPVs chronically excreted by persons with certain forms of primary immunodeficiencies [PID]). When OPV is accidentally administered to an immunodeficient individual, there is a risk of prolonged virus excretion, potentially initiating an outbreak (note that no iVDPV-related outbreaks have been conclusively attributed to iVDPV excretors to date). The SAGE recommended that a specialized surveillance system for PID patients and iVDPV be integrated into routine polio environmental and AFP surveillance [51]. Thus far, PID surveillance is being implemented in twelve countries as pilot initiatives, research projects, or related to nOPV2 EUL requirements [17]. Additionally, there are several R&D projects to pursue effective antiviral drugs to reduce excretion and risk of paralysis in iVDPV-infected PID patients, including V-7404 and pocapavir as potential candidates led by the Poliovirus Antivirals Initiative (PAI) [52]. Identification and management of iVDPV are essential in the post-certification era, as it could be the only persistent reservoir (aside from manufacturing facilities) of poliovirus after eradication and OPV withdrawal.

Current manufacturing of IPV vaccines requires the use of large quantities of live poliovirus (either WPV for production of conventional IPV or Sabin poliovirus for production of sIPV). Efforts are being made to produce IPV in a process that will not involve the use of live poliovirus. A virus-like particle (VLP) platform for the production of IPV-like vaccines is being developed, with CanSino, China ready to start in-human clinical trials and multiple other candidates in the preclinical phase [53,54]. The goal is to have a VLP product on the market in late 2020s. In addition to polio VLPs and following the WHO’s recommendation to produce IPVs using attenuated strains, IPV is being developed using S19 virus strains developed by MHRA (formerly NIBSC, UK). The S19 strain has been modified so that it is unlikely to replicate in humans, so it has obtained a containment waiver [55]. Currently, an S19 hexavalent vaccine from Biological E, India is in phase I clinical study. 

### 4.2. Polio Vaccination in the Post-Certification Era

Current SAGE recommendations call for continuation of vaccinations with IPV for at least 10 years after the last poliovirus type is certified as eradicated and indefinitely in countries hosting facilities handling live polioviruses [56]. It is, however, anticipated that some countries, especially those using hexavalent vaccines, will continue to immunize their populations against polio indefinitely.

## 5. Concluding Remarks

The historical narrative and persisting work towards polio eradication underscore the challenges, failures, and successes of this tremendous global effort. Since there is no cure for poliomyelitis, the strategic delivery of polio vaccination through all its forms (routine immunization programs, supplementary polio campaigns, injectables, oral administration, etc.) is the most effective method to achieve eradication. Moreover, research and development with respect to novel tools, including diagnostic methods, vaccines, antivirals, and outreach initiatives, are essential for rapid notification of outbreaks and sustainable vaccination. 

There are many unforeseen challenges in the polio endgame. Geopolitical risks directly impact eradication efforts, particularly through conflict, insecurity, and inaccessibility of certain populations. Poliovirus serotype co-circulation increases the complexity of outbreak response, and competing public health priorities require available resources to be shared. Nonetheless, historically endemic reservoirs of WPV1 are disappearing, and the genetic diversity is markedly reduced to just two chains of transmission in one epidemiological block. Surveillance indicators and vaccination coverage have steadily recovered and surpassed pre-COVID-19 pandemic levels. 

The eradication endgame is incredibly complex, and successes are fragile. Eradicating the final chains of WPV1 and cVDPV transmission and preventing more children from becoming paralyzed by poliovirus will require renewed financial and political commitments from governments, donors, multilateral organizations, and local communities from across the world.
